# Aligning genotyping and copy number data in single trophectoderm biopsies for aneuploidy prediction: uncovering incomplete concordance

**DOI:** 10.1093/hropen/hoae056

**Published:** 2024-09-18

**Authors:** Lisa De Witte, Machteld Baetens, Kelly Tilleman, Frauke Vanden Meerschaut, Sandra Janssens, Ariane Van Tongerloo, Virginie Szymczak, Dominic Stoop, Annelies Dheedene, Sofie Symoens, Björn Menten

**Affiliations:** Center for Medical Genetics, Department of Biomolecular Medicine, Ghent University, Ghent, Belgium; Center for Medical Genetics, Ghent University Hospital, Ghent, Belgium; Department for Reproductive Medicine, Ghent University Hospital, Ghent, Belgium; Department for Reproductive Medicine, Ghent University Hospital, Ghent, Belgium; Center for Medical Genetics, Ghent University Hospital, Ghent, Belgium; Center for Medical Genetics, Ghent University Hospital, Ghent, Belgium; Center for Medical Genetics, Ghent University Hospital, Ghent, Belgium; Department for Reproductive Medicine, Ghent University Hospital, Ghent, Belgium; Center for Medical Genetics, Ghent University Hospital, Ghent, Belgium; Center for Medical Genetics, Ghent University Hospital, Ghent, Belgium; Center for Medical Genetics, Department of Biomolecular Medicine, Ghent University, Ghent, Belgium; Center for Medical Genetics, Ghent University Hospital, Ghent, Belgium

**Keywords:** preimplantation genetic testing, PGT, aneuploidy, chromosomal abnormalities, chromosomal mosaicism, copy number, genotyping, meiosis, mitosis, monogenic disease

## Abstract

**STUDY QUESTION:**

To what extent can genotype analysis aid in the classification of (mosaic) aneuploid embryos diagnosed through copy number analysis of a trophectoderm (TE) biopsy?

**SUMMARY ANSWER:**

In a small portion of embryos, genotype analysis revealed signatures of meiotic or uniform aneuploidy in those diagnosed with intermediate copy number changes, and signatures of presumed mitotic or putative mosaic aneuploidy in those diagnosed with full copy number changes.

**WHAT IS KNOWN ALREADY:**

Comprehensive chromosome screening (CCS) for preimplantation genetic testing has provided valuable insights into the prevalence of (mosaic) chromosomal aneuploidy at the blastocyst stage. However, diagnosis of (mosaic) aneuploidy often relies solely on (intermediate) copy number analysis of a single TE biopsy. Integrating genotype information allows for independent assessment of the origin and degree of aneuploidy. Yet, studies aligning both datasets to predict (putative mosaic) aneuploidy in embryos remain scarce.

**STUDY DESIGN, SIZE, DURATION:**

A single TE biopsy was collected from 1560 embryos derived from 221 couples tested for a monogenic disorder (n = 218) or microdeletion-/microduplication syndrome (n = 3). TE samples were subjected to both copy number and genotyping analysis.

**PARTICIPANTS/MATERIALS, SETTING, METHODS:**

Copy number and SNP genotyping analysis were conducted using GENType. Unbalanced chromosomal anomalies ≥10 Mb (or ≥20 Mb for copy number calls <50%) were classified by degree, based on low-range intermediate (LR, 30–50%), high-range intermediate (HR, 50–70%) or full (>70%) copy number changes. These categories were further subjected to genotyping analysis to ascertain the origin (and/or degree) of aneuploidy. For chromosomal gains, the meiotic division of origin (meiotic I/II versus non-meiotic or presumed mitotic) was established by studying the haplotypes. The level of monosomy (uniform versus putative mosaic) in the biopsy could be ascertained from the B-allele frequencies. For segmental aneuploidies, genotyping was restricted to deletions.

**MAIN RESULTS AND THE ROLE OF CHANCE:**

Of 1479 analysed embryos, 24% (n = 356) exhibited a whole-chromosome aneuploidy, with 19% (n = 280) showing full copy number changes suggestive of uniform aneuploidy. Among 258 embryos further investigated by genotyping, 95% of trisomies with full copy number changes were identified to be of meiotic origin. For monosomies, a complete loss of heterozygosity (LOH) in the biopsy was observed in 97% of cases, yielding a 96% concordance rate at the embryo level (n = 248/258). Interestingly, 4% of embryos (n = 10/258) showed SNP signatures of non-meiotic gain or putative mosaic loss instead. Meanwhile, 5% of embryos (n = 76/1479) solely displayed HR (2.5%; n = 37) or LR (2.6%; n = 39) intermediate copy number changes, with an additional 2% showing both intermediate and full copy number changes. Among embryos with HR intermediate copy number changes where genotyping was feasible (n = 25/37), 92% (n = 23/25) showed SNP signatures consistent with putative mosaic aneuploidy. However, 8% (n = 2/25) exhibited evidence of meiotic trisomy (9%) or complete LOH in the biopsy (7%). In the LR intermediate group, 1 of 33 (3%) genotyped embryos displayed complete LOH. Furthermore, segmental aneuploidy was detected in 7% of embryos (n = 108/1479) (or 9% (n = 139) with added whole-chromosome aneuploidy). These errors were often (52%) characterized by intermediate copy number values, which closely aligned with genotyping data when examined (94–100%).

**LARGE SCALE DATA:**

N/A.

**LIMITATIONS, REASONS FOR CAUTION:**

The findings were based on single TE biopsies and the true extent of mosaicism was not validated through embryo dissection. Moreover, evidence of absence of a meiotic origin for a trisomy should not be construed as definitive proof of a mitotic error. Additionally, a genotyping diagnosis was not always attainable due to the absence of a recombination event necessary to discern between meiotic II and non-meiotic trisomy, or the unavailability of DNA from both parents.

**WIDER IMPLICATIONS OF THE FINDINGS:**

Interpreting (intermediate) copy number changes of a single TE biopsy alone as evidence for (mosaic) aneuploidy in the embryo remains suboptimal. Integrating genotype information alongside the copy number status could provide a more comprehensive assessment of the embryo’s genetic makeup, within and beyond the single TE biopsy. By identifying meiotic aberrations, especially in presumed mosaic embryos, we underscore the potential value of genotyping analysis as a deselection tool, ultimately striving to reduce adverse clinical outcomes.

**STUDY FUNDING/COMPETING INTEREST(S):**

L.D.W. was supported by the Research Foundation Flanders (FWO; 1S74621N). M.B., K.T., F.V.M., S.J., A.V.T., V.S., D.S., A.D., and S.S. are supported by Ghent University Hospital. B.M. was funded by Ghent University. The authors have no conflicts of interest.

WHAT DOES THIS MEAN FOR PATIENTS? Chromosomal abnormalities play a significant role in miscarriages and birth defects in humans.

When patients undergo preimplantation genetic testing (PGT) to address fertility issues, their embryos are screened for these abnormalities.

Typically, embryos are only examined for extra or missing chromosomes in a single biopsy, known as copy number analysis. The degree of copy number change is then used to estimate whether the embryo is entirely normal or abnormal or whether it contains a mix of cells with different chromosomal content, a phenomenon called mosaicism. This, in turn, affects the decision of whether or not to transfer the embryo to the mother. However, diagnosing mosaicism based solely on this copy number approach is not always accurate due to biological and technical limitations, which can lead to misclassification of embryos. This research provides insight into how a second strategy, called genotype analysis, can aid the embryo (de)selection process of PGT. By independently assessing the origin (and degree) of chromosomal abnormalities, this method can help to identify embryos that are less likely to result in a successful pregnancy.

## Introduction

Cytogenetic abnormalities have been recurrently identified in tissues from first-trimester miscarriages following spontaneous conception and IVF (Pylyp *et al.*, 2018). Among etiopathogenic factors, chromosomal abnormalities account for approximately 50% of early pregnancy losses (Martínez *et al.*, 2010; Pylyp *et al.*, 2018; Wu *et al.*, 2021). On the other hand, those proven to be viable can lead to severe congenital disorders in live-born infants (Wellesley *et al.*, 2012). To maximize the patient’s chance of having a healthy baby, efforts have been made to integrate comprehensive chromosome screening (CCS) during preimplantation genetic testing (PGT). Accordingly, in both clinical and research context, this approach has yielded substantial cytogenetic data at early developmental stages, especially in patients enrolled for aneuploidy screening (PGT-A) (Fragouli *et al.*, 2019).

Still, the cytogenetic assessment remains incomplete. Over the years, most technologies used to study the genomic landscape of embryos have relied solely on copy number analysis (e.g. quantitative polymerase chain reaction, fluorescence *in situ* hybridization, array comparative genomic hybridization, or low-coverage whole-genome sequencing) (Deleye *et al.*, 2015; Kung *et al.*, 2015; Chen *et al.*, 2020). While valuable, such technologies preclude analysis of the origin of aneuploidies, even though the clinical repercussions might be vastly different. Whereas meiotic errors are assumed to affect all embryonic cells and are unequivocally harmful, mitotic errors lead to embryo mosaicism, which has been linked with healthy live births, albeit with lower success rates than with euploid embryos (Greco *et al.*, 2015; Spinella *et al.*, 2018; Munné *et al.*, 2020; Capalbo *et al.*, 2021; Viotti *et al.*, 2021b). Recognizing the phenomenon of chromosomal mosaicism and its recently established clinical potential, the sole classification of embryos based on a bimodal reporting system (i.e. normal or abnormal) might not be sustainable, though this remains a topic of debate (Treff and Marin, 2021; Campos, 2023; Girardi *et al.*, 2023).

Establishing the true extent of chromosomal mosaicism at early stages remains challenging. Currently, intermediate copy number changes of a single trophectoderm (TE) biopsy alone are interpreted as evidence of a mixture of euploid and aneuploid cells, and therefore diagnosed as mosaicism. However, the low consensus in reporting strategies (e.g. selected copy number thresholds chosen for mosaicism reporting) and testing strategies (e.g. analytical algorithms) has created substantial discrepancies in the prevalence reported (Popovic *et al.*, 2020, 2024; Girardi *et al.*, 2023). Meanwhile, the inability to distinguish true biological signals from technical artifacts superimposed on euploid and aneuploid embryos casts further doubt. Additionally, the phenomenon of mosaicism itself challenges the role of a single TE biopsy and its copy number as representative (Viotti *et al.*, 2021a). Even when the copy number accurately reflects the content of the TE biopsy, it might not reflect the chromosomal status of the embryo, and especially the inner cell mass, due to sampling bias (Popovic *et al.*, 2020; Treff and Marin, 2021). Thus, investigation of origins may be imperative to estimate the embryo’s full genetic spectrum, and ultimately, its true clinical potential (Handyside *et al.*, 2021; Rana *et al.*, 2023). In this pursuit, genome-wide haplotyping strategies have emerged as a valuable tool, leveraging genotyping data to unveil the meiotic or (presumed) mitotic origin of a trisomy, assess the degree of monosomy in the biopsy, and identify the parental origin (Handyside *et al.*, 2010; Zamani Esteki *et al.*, 2015; De Witte *et al.*, 2022; Masset *et al.*, 2022). Moreover, genotyping facilitates the detection of genome-wide ploidy or copy-neutral anomalies, which are hidden to copy number analysis.

Here, we study the prevalence of common and rare (putative mosaic) chromosomal anomalies in human blastocysts using the genome-wide haplotyping-based pipeline ‘GENType’ (De Witte *et al.*, 2022). Striving for more rigorous classification by degree (i.e. uniform aneuploidy versus putative mosaicism), we examined both copy number data and genotyping data in parallel, providing a concordance rate between both datasets. Altogether, our data offers valuable insights for refining the current embryo (de)selection process of PGT.

## Materials and methods

### Study cohort

The study cohort consisted of 1560 blastocysts, derived from 311 oocyte collection cycles, undergoing comprehensive PGT at the Department for Reproductive Medicine and Center for Medical Genetics of Ghent University Hospital from April 2021 to January 2023. All blastocysts originated from couples (n = 221), with a normal karyotype, burdened by a monogenic disorder (PGT-M) (couples: n = 218, disorders: n = 125) or a microdeletion-/microduplication syndrome (couples: n = 3, disorders: n = 2). Maternal age ranged from 22 to 44 with a mean of 31 years (Supplementary Fig. S1). To enable comprehensive SNP analysis of the embryos, parental DNA (of one or both biological parents) and DNA from phasing reference(s) (grandparent(s)/sibling of the embryo) were retrieved, if available.

### Ethical approval

The study was approved by the Institutional Review Board of Ghent University Hospital (reference number: ONZ-2022-0044). All couples provided informed consent on the use of their data.

### Embryo biopsy

Human blastocysts were individually cultured in 25 μl micro drops in sequential medium from either Cook Medical (Limerick, Ireland) under mineral oil (FUJIFILM Irvine Scientific, Tilburg, The Netherlands) or sequential medium from Origio (Malov, Denmark) under liquid paraffin oil (Origio). Laser assisted hatching (LYKOS laser, Hamilton Thorne, Beverly, MA, USA) was performed on day 4 or 5 of development. Following embryo grading according to the Gardner and Schoolcraft grading criteria, hatching blastocysts with a minimum score of grade C for inner cell mass (ICM) and TE underwent laser-assisted biopsy (Zilos-TK, Hamilton Thorne). A single TE biopsy (approximately 5–10 cells) was obtained from each embryo. The biopsied cells were washed with phosphate-buffered saline (VWR, Leuven, Belgium) and 10% (w/v) polyvinylpyrrolidone (Origio) and placed in 0.2 ml sterile PCR tubes. Samples were kept at −20°C until genetic testing.

### Whole-genome amplification

Biopsies were subjected to whole-genome amplification (WGA) using the PicoPLEX Single Cell WGA kit V3 (Takara Bio, Saint-Germain-en-Laye, France) or the REPLI-g single cell kit (QIAGEN, Hilden, Germany). To remove residual primers, PicoPLEX products were purified using Agencourt Ampure XP beads (Beckman Coulter, Brea, CA, USA). WGA concentrations were measured by the QubitTM dsDNA (Broad-Sensitivity) assay kit (Thermo Fisher Scientific, Merelbeke, Belgium) and fragment size was determined by fragment analyser qualitative 915/930 dsDNA kits (Agilent technologies, Santa Clara, CA, USA). WGA samples characterized by low DNA yield (<100 ng) were not processed further as this is an indication for low quality amplification. Both WGA kits were validated for genetic analysis (De Witte *et al.*, 2022).

### GENType

All TE biopsies were analysed by GENType, a reduced representation sequencing (RRS)-based technology that allows genome-wide haplotyping as well as copy number analysis. GENType has been extensively validated as described by De Witte *et al.* (2022). Libraries were fully automatically prepared on a Hamilton Star liquid handler (Hamilton Company, Reno, NV, USA) and equimolar concentrations were pooled for paired-end sequencing (2 × 150 cycles) on a NovaSeq 6000 Sequencing system (Illumina, San Diego, CA, USA) at a minimum of 25M read pairs per sample.

### Combining copy number and SNP genotyping analysis

**Figure 1.**
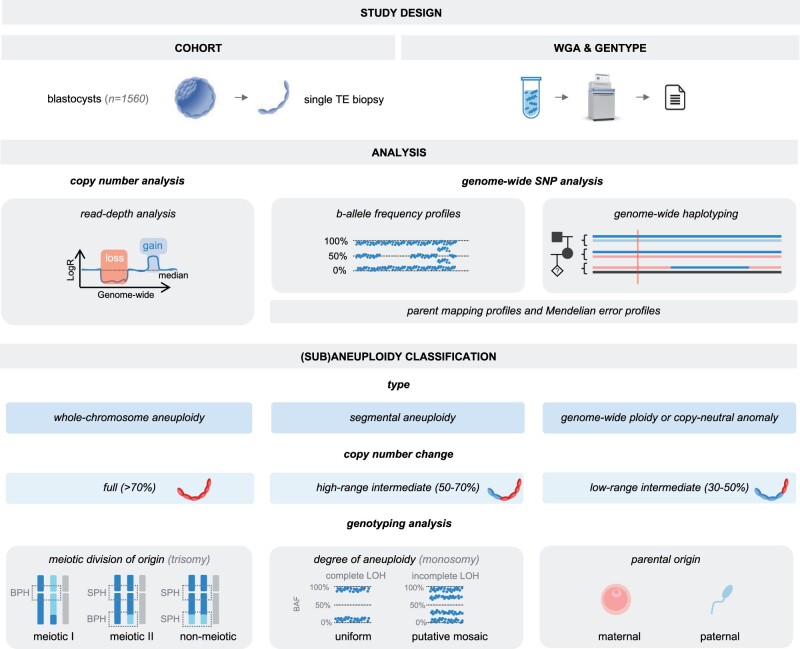
**Graphic overview of study design.** Single TE biopsies from PGT-M blastocysts were analysed by GENType for concurrent copy number and genotype analysis. Detected genomic abnormalities were classified by type and copy number change (full: >70%, HR intermediate: 50–70%, LR intermediate: 30–50%). Anomalies underwent further genotyping analysis to ascertain their origin and degree. The meiotic division of origin for trisomies was established by studying haplotypes: BPH in the centromere indicated meiotic I trisomy, while BPH or SPH by a recombination event indicated meiotic II and non-meiotic (or presumed mitotic) trisomy, respectively. Monosomy degree was ascertained using BAF, distinguishing uniform zygosity (complete LOH) from putative mosaic zygosity (incomplete LOH). Concordance between copy number and genotyping analysis was outlined on chromosome and embryo level. The parental nature (i.e. maternal, paternal) of anomalies (with copy number changes >50%) was also determined. TE, trophectoderm; WGA, whole-genome amplification; HR, high range; LR, low range; BPH, both parental homologs; SPH, single parental homologue; BAF, b-allele frequencies; LOH, loss of heterozygosity.

All TE samples underwent copy number analysis using WisecondorX with visualization through ViVar (Sante *et al.*, 2014; Raman *et al.*, 2019), and SNP genotyping with Hopla (De Witte *et al.*, 2022) (Fig. 1). Copy number change was inferred from Log_2_ transformed ratios, representing the observed number of reads over the expected number of reads. Genotyping analysis involved examining haplotypes, SNP heterozygosity patterns (e.g. parent mapping profiles and Mendelian error profiles) and b-allele frequencies (BAF). Embryos with copy number changes <30% and regular SNP distributions were deemed euploid. Embryos with chromosomal abnormalities ≥10 Mb (or ≥20 Mb for copy number changes <50%) were further classified by aberration type. Log_2_R profiles exhibiting excessive noise or a wavy outline upon visual inspection (most likely attributed to WGA artifacts, lower embryonal quality or lower biopsy quality) were excluded from further analysis.

To assess the degree of aneuploidy (i.e. uniform aneuploidy versus putative mosaicism), two sources of data were combined. First, aberrations were classified after copy number analysis into three groups: low-range intermediate (LR, 30–50%), high-range intermediate (HR, 50–70%) or full (>70%) copy number changes. Second, SNP genotyping was performed on these aneuploidies. Chromosomal gains (e.g. trisomies and triploidy) underwent a meiotic division of origin analysis based on haplotypes and SNP heterozygosity patterns, as per validation (De Witte *et al.*, 2022). A meiotic gain was identified through the presence of both parental homologs (BPH) of the contributing parent across the centromere (meiotic I (MI) error) or other parts of the chromosome (meiotic II (MII) error) (Ariad *et al.*, 2021). Conversely, copy number increases composed of a single parental homologue (SPH) were considered of non-meiotic (or presumed mitotic) origin (Fig. 1). It is important to note that we refrain from assuming the evidence of absence of a meiotic origin as definitive evidence of a mitotic error. Without additional evidence of a mitotic error, the presence of an SPH tract was considered insufficient to distinguish genuine copy number changes from technical artifacts superimposed on euploid profiles. Moreover, distinction between MII and non-meiotic errors was only feasible when a crossover event occurred in the trisomic chromosome. For this reason, copy number gains including an SPH without a recombination event were excluded from origin analysis. For chromosomal losses, we differentiated uniform from putative mosaic zygosity in the TE biopsy through the study of the BAF patterns (De Witte *et al.*, 2022). BAF levels of 0% and 100% (i.e. complete loss of heterozygosity (LOH)) constituted evidence of uniform zygosity in the TE biopsy. In contrast, copy loss combined with SNP heterozygosity with low or high variant allele frequencies (i.e. BAF levels between 0–50% and 50–100%), which we herein refer to as incomplete LOH, implied putative mosaic zygosity in the biopsy. Further differentiation of mosaicism degrees through BAF analysis could not be conducted due to technical limitations.

Concordance between copy number and genotyping data was calculated at both the chromosome and embryo level. Aneuploidies with full copy number changes, indicative of uniform aneuploidy, were expected to be meiotic in origin (for trisomies) or to exhibit complete LOH in the TE biopsy (for monosomies). Aneuploidies with intermediate copy number changes, suggestive of mosaic aneuploidy, were expected to be of non-meiotic (or presumed mitotic) origin or to display incomplete LOH in the TE biopsy.

We further unraveled the parental nature (i.e. maternal or paternal) of all chromosomal abnormalities with copy number changes >50%, with the exception of non-meiotic trisomies due to software limitations. For segmental aneuploidies, genotype analysis solely focused on deletions, due to the limited resolution of parent mapping for duplications (De Witte *et al.*, 2022). Overall, genotype analysis (except for BAF analysis) was contingent on the availability of DNA from both parents. Samples failing quality control metrics (e.g. low SNP call rate, allelic drop-out rate >50%) were excluded from genotyping analysis.

### Validation of intermediate copy number detection by GENType

To validate GENType for accurate detection of intermediate copy number changes, a series of cell mixing experiments were conducted. By combining aneuploid cells with euploid cells at different ratios, mosaicism was simulated for both a whole-chromosome aneuploidy (trisomy 21, GM04927) and a segmental aneuploidy (15 Mb terminal 18q deletion, GM11951). Individual cells from the different aneuploid cell lines and a euploid cell line (GM12885 (46,XX)) were collected through micromanipulation. In total, 10 cells were mixed to reflect the sample size of a TE biopsy typically conducted at our PGT centre, as per the following aneuploid:euploid ratios: 10:0; 7:3; 5:5; 3:7; 0:10. All samples underwent WGA and GENType as described above. Each mosaic mixing experiment was performed in quadruplicate (i.e. in duplicate for both applied WGA methods), except for the 0:10 ratio (no replicates due to entirely euploid mix). A linear correlation between the LogR values (and consequently copy number status) and the number of abnormal cells was observed for both whole-chromosome aneuploidies (i.e. trisomy 21 and a monosomy X simulated by different genders of the cell lines) and segmental aneuploidy (Supplementary Fig. S2).

### Statistical analysis

Statistical analysis was performed using GraphPad Prism (GraphPad Software, San Diego, CA, USA, version 9). Categorical variables were compared using a Chi-square test (for trend). A binomial test was used in the event of two possible outcomes. Statistical significance was defined by a *P* value less than 0.05.

## Results

### General data

A total of 1560 embryos were biopsied. Successful WGA was achieved for 1524 (98%) embryos. The remaining TE biopsies (n = 36; 2%) either failed to amplify (n = 24) or displayed suboptimal amplification (showing allelic drop-out rates >85%) (n = 12), most likely due to low biopsy or sample quality, given the established performance of both WGA kits (De Witte *et al.*, 2022). Quality assessment of the successfully amplified samples revealed a mean allelic drop-out rate of 18.6% and allelic drop-in rate of 1.57% across a genome-wide marker density of approximately 102 000 SNPs/genome, which is in line with our validation study (De Witte *et al.*, 2022). Only one sample (0.06%) was excluded due to complete maternal contamination, therefore our study cohort included a total of 1523 samples.

### Prevalence and type of genomic anomalies in blastocysts

All embryos were subjected to CCS. For 1479 out of 1523 embryos (97%), an interpretable copy number analysis result could be obtained (after visual inspection of Log_2_R profiles). Of these, 1012 embryos (68%) were euploid, while 467 embryos (32%) contained one or multiple (sub)chromosomal abnormalities. Whole-chromosome aneuploidy was the most observed event (22%), followed by segmental aneuploidy (7%), and a combination of both (2%) (Table 1).

**Table 1.** Chromosomal anomalies detected in PGT-M blastocysts.

Ploidy status	Total embryos n (%)	**Embryos by chromosomal anomaly count** ^a^ **n (%)**			
		*Single*	*Double*	*Multiple (3–4)*	*Complex (≥5)*
Euploid	1012 (68%)				
Abnormal	467 (32%)				
Whole-chromosome aneuploidy^b^	325 (22%)	*217 (15%)*	*65 (4%)*	*25 (2%)*	*18 (1%)*
Segmental aneuploidy^b^	108 (7%)	*78 (5%)*	*29 (2%)*	*1 (0.1%)*	*0 (0%)*
Whole-chromosome and segmental aneuploidy	31 (2%)	*26 (1.8%)^c^*	*4 (0.3%)^c^*	*0 (0%)^c^*	*1 (0.1%)^c^*
Genome-wide ploidy anomaly	3 (0.2%)				
Copy-neutral anomaly	0 (0%)				

Each percentage represents the proportion of the 1479 embryos investigated.

a The number (and proportion) of embryos exhibiting single, double, multiple, or complex aneuploidy (whole-chromosome or segmental). For embryos with <5 anomalies in conjunction, each aneuploidy was studied as a singular event for copy number and genotyping analysis at the chromosome level.

b Exclusively this type of chromosomal anomaly detected in the embryo.

c In this group, the number of reported anomalies exclusively reflect the quantity of whole-chromosome aneuploidy within the embryo, disregarding additional segmental aneuploidy.

### Distribution of whole-chromosome aneuploidy by copy number change

**Figure 2.**
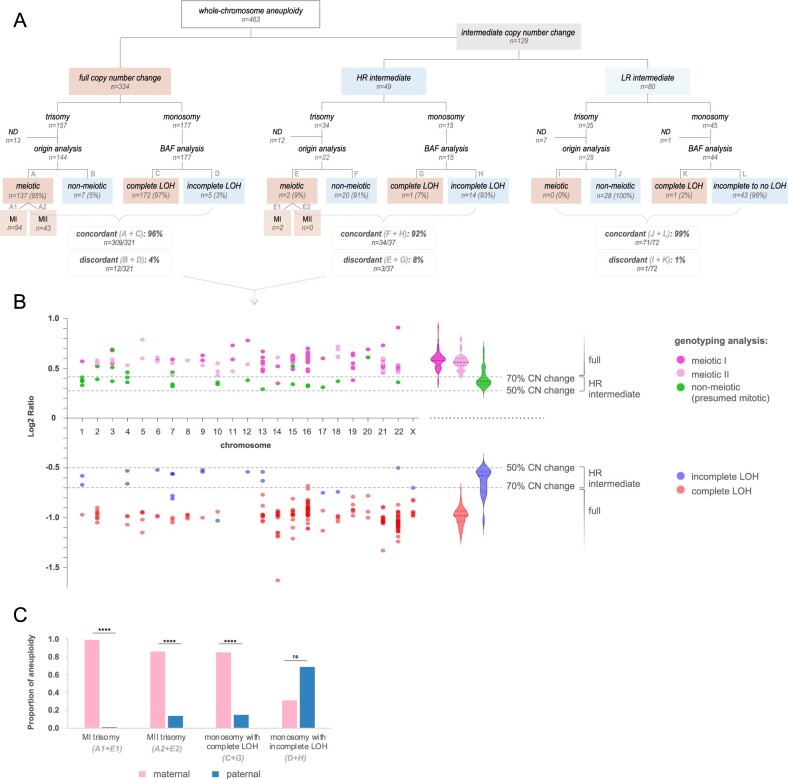
**Characterization of whole-chromosome aneuploidy according to copy number change and genotyping analysis.** (**A**) Proportion of whole-chromosome aneuploidies per copy number change (upper branch) and genotyping analysis (lower branch). Those not investigated by genotyping are categorized as ND. Concordance between both datasets is shown at the bottom of each branch. (**B**) Scatterplot (on the left) displaying copy number and genotype results for individual whole-chromosome aneuploidies, organized per chromosome (*x*-axis). Each data point represents a distinct whole-chromosome aneuploidy, and only data points within the full and HR intermediate copy number categories are shown. Log2 ratios are presented on the *y*-axis, with dashed lines marking the copy number change thresholds (i.e. 50–70% for HR intermediate copy number change and >70% for full copy number change). Data points are colour-coded by genotype result: meiotic I (dark pink), meiotic II (light pink), non-meiotic (or presumed mitotic) (green), complete LOH (red), incomplete LOH (blue). Violin plots (in the middle) show the frequency distribution of Log2 ratios for each genotype group. The width of the violin plot corresponds to data density, with the middle line representing the median, and the top and bottom lines, representing the 75th and 25th percentiles, respectively. (**C**) Parental origin of whole-chromosome aneuploidy classified according to genotyping diagnosis. BAF, b-allele frequencies; CN, copy number; ND, not determined; HR, high range; LR, low range; LOH, loss of heterozygosity; MI, meiotic I; MII, meiotic II.

To study chromosomal instability, whole-chromosome aneuploidies from embryos (with <5 aberrations in conjunction) were analysed as singular events for further analysis. In total, 463 whole-chromosome aneuploidies were studied (Fig. 2). Of these, the majority (n = 334; 72%) demonstrated full copy number changes. Still, over a quarter (n = 129; 28%) of the aneuploidies displayed intermediate copy number changes, with a slightly lower prevalence showing HR values than LR values (Fig. 2A).

Whole-chromosome aneuploidies with full copy number changes were significantly more frequently detected in acrocentric and small chromosomes (*P *<* *0.0001; *χ*^2^ test), with a predominance of errors in chromosome 16 and 22 (Supplementary Fig. S3A and B). In contrast, aneuploidies with intermediate copy number changes, indicative for chromosomal mosaicism, were scattered across the chromosomes with no significant preference towards a particular chromosomal size (*P *>* *0.05; *χ*^2^ test) (Supplementary Fig. S3A and B). This distribution pattern was consistent with the genotype signatures observed for each chromosome category. SNP signatures indicative of uniform aneuploidy (i.e. meiotic trisomy, monosomy with complete LOH) were predominantly noted in acrocentric and small chromosomes (*P *<* *0.0001; *χ*^2^ test), while signatures indicative of putative mosaicism (i.e. non-meiotic trisomy, monosomy with incomplete LOH) showed no significant correlation with chromosome length (*P *>* *0.05; *χ*^2^ test) (Supplementary Fig. S3C).

### Concordance between copy number and genotyping data for whole-chromosome aneuploidy

A detailed investigation of SNP genotyping data for each copy number category was conducted. An overview of the results is given in Fig. 2A and B. Any discrepancies found between the copy number analysis and the genotype analysis are presented in Table 2, providing the specifics on the chromosomes involved.

**Table 2.** Discrepancies in copy number change and genotyping diagnosis for whole-chromosome aneuploidy.

Embryo	Chromosome	Copy number category	Copy number change	SNP genotyping diagnosis
1	Trisomy 2	Full	90%	Non-meiotic
2	Trisomy 3	Full	87%	Non-meiotic
3	Trisomy 7	Full	79%	Non-meiotic
4	Trisomy 4	Full	80%	Non-meiotic
	Trisomy 15	Full	89%	Non-meiotic
	Trisomy 20	Full	103%	Non-meiotic
5	Trisomy 3	Full	114%	Non-meiotic
	*(2p dup, 2q del)*	*(full, full)*	*(105%,133%)*	*(ND, complete LOH)*
6	Monosomy 7	Full	76%	Incomplete LOH
7	Monosomy 10	Full	99%	Incomplete LOH
8	Monosomy 17	Full	73%	Incomplete LOH
9	Monosomy 18	Full	72%	Incomplete LOH
10	Monosomy 7	Full	78%	Incomplete LOH
	*(3q dup)*	*(LR intermediate)*	*(50%)*	*(ND)*
11	Trisomy 19	HR intermediate	66%	Meiotic I
	*(monosomy 20)*	*(full)*	*(96%)*	*(complete LOH)*
12	Trisomy 14	HR intermediate	61%	Meiotic I
	*(trisomy 6, trisomy 18)*	*(LR intermediate, LR intermediate)*	*(39%, 41%)*	*(non-meiotic, non-meiotic)*
13	Monosomy 16	HR intermediate	66%	Complete LOH
	*(4q dup)*	*(HR intermediate)*	*(63%)*	*(ND)*
14	Monosomy 8	LR intermediate	43%	Complete LOH
	*(monosomy 17)*	*(LR intermediate)*	*(31%)*	*(incomplete to no LOH)*

Additional aberrations in the embryo are noted within parentheses in italics.

ND, not determined; LOH, loss of heterozygosity; HR, high range; LR, low range.

Among the whole-chromosome aneuploidies displaying full copy number changes (n = 334), the majority (n = 321/334; 96%) underwent SNP analysis. This included most trisomies (n = 144/157; 92%) and all monosomies (n = 177/177). The few trisomies (n = 13; 8%) excluded from analysis were due to single-parent processing (n = 7/13), poor quality (i.e. low SNP call rate) (n = 1/13), or the absence of a recombination in the trisomic chromosome (n = 5/13) due to the associated inability to distinguish MII from mitotic errors.

Of the analysed trisomies with full copy number changes, approximately 95% (n = 137/144) were of corresponding meiotic origin, with an increased rate of MI errors (n = 94/144; 65%) over MII errors (n = 43/144; 30%, Supplementary Fig. S4A). Surprisingly, 5% (n = 7/144) turned out to be of non-meiotic (and hence presumed mitotic) origin (Supplementary Fig. S4C). A similar concordance between both data sources was observed for monosomies. Here, prediction of uniform monosomy by copy number was reinforced by complete LOH in the TE biopsy in 97% of cases (n = 172/177, Supplementary Fig. S5A). In 3% (n = 5/177), however, incomplete LOH could be detected (Supplementary Fig. S5C).

In summary, most aneuploidies (n = 309/321; 96%) diagnosed with a copy number above 70% showed SNP signatures consistent with uniform aneuploidy in the biopsy/embryo, while evidence of uniform aneuploidy was lacking in a small subset (n = 12/321; 4%) (Fig. 2B).

Within the category of whole-chromosome aneuploidies with HR intermediate copy number changes (n = 49), SNP information could be obtained for 22 out of 34 trisomies (65%) and all 15 monosomies. Trisomies (n = 12/34) lacking diagnosis were attributed to single-parent analysis (n = 2/12), an inconclusive result (n = 1/12), or the absence of a crossover in the trisomic chromosome (n = 6/12). For the remaining cases (n = 3/12), SNP analysis revealed partial maternal contamination, obscuring the SNP signatures.

Trisomies with HR intermediate copy number changes, subjected to further analysis, were primarily of non-meiotic (or presumed mitotic) origin (n = 20/22; 91%, Supplementary Fig. S4B), although not exclusively. Unexpectedly, 2 out of 22 trisomies (9%) diagnosed as putative mosaic by intermediate copy number turned out to be of meiotic (I) origin (Supplementary Fig. S4D). As for monosomies, prediction of putative mosaicism by HR intermediate copy number loss corresponded with incomplete LOH for 93% of the cases (n = 14/15, Supplementary Fig. S5B). On the other hand, complete LOH in the biopsy was observed for 7% (n = 1/15) of the supposed intermediate copy number losses (Supplementary Fig. S5D).

In summary, most whole-chromosome aneuploidies (n = 34/37; 92%) with HR intermediate copy number changes displayed SNP signatures consistent with putative mosaicism. However, a considerable portion of errors (n = 3/37; 8%) exhibited evidence of meiotic or uniform aneuploidy in the biopsy instead (Fig. 2B).

Finally, for the LR intermediate copy number group, genotype signatures were mapped for most trisomies (n = 28/35) and monosomies (n = 44/45). The remaining cases were excluded due to similar reasons (i.e. absence of a crossover in the trisomic chromosome: n = 5, inconclusive result: n = 1, single-parent processing: n = 1, partial maternal contamination: n = 1). All analysed trisomies were of non-meiotic origin, whereas 1 monosomy displayed complete LOH (Supplementary Fig. S5E). This concluded the concordance rate at 99% at the chromosome level.

### Parental nature of whole-chromosome aneuploidy

Next, we investigated the parental nature of whole-chromosome aneuploidies (Fig. 2C). We examined all trisomies of meiotic origin (detected across the full and HR intermediate copy number categories) (n = 139) and the majority of monosomies characterized by complete LOH (n = 167/173) or incomplete LOH (n = 16/19) (across the same two copy number categories). The remaining monosomies were not considered due to single-parent processing (n = 6/173) or inconclusive outcomes (n = 3/19).

Meiotic trisomies were primarily of maternal origin (*P *<* *0.0001, binomial test) (n = 132/139; 95%). In fact, all MI errors seemed to be maternally derived, except for one paternal MI error (n = 95/96; 99%). Among MII errors, 86% (n = 37/43) showed signatures of maternal gain and 14% (n = 6/43) of paternal gain. A similar distribution was observed for monosomies with complete LOH in the biopsy, with a prominent prevalence of maternal origin (*P *<* *0.0001, binomial test) (n = 142/167, 85%), suggesting a meiotic origin. Altogether, maternal origin was predominant for whole-chromosome aneuploidy believed to be uniformly present in the embryo (n = 274/306; 90%). Correspondingly, the incidence of these anomalies displayed an upward trend with maternal age (*P *<* *0.0001; *χ*^2^ test for trend) (Supplementary Fig. S3D).

Mitotic errors, on the other hand, are not expected to discriminate between maternal and paternal homologs. Indeed, no significant correlation (*P *>* *0.05; binomial test) between parental origin and the rate of monosomy with incomplete LOH (suggestive of mitotic errors) was observed, despite the apparent preference for the paternal allele (maternal origin: n = 5/16; paternal origin: n = 11/16). The apparent bias towards the paternal allele is questionable and should be interpreted with caution due to limited data and comparatively substantial numbers of unknown origin (n = 3/19). In contrast to meiotic aneuploidy, no correlation with maternal age could be observed for whole-chromosome aneuploidy characterized by intermediate copy number changes and non-meiotic/putative mosaic SNP signatures (*P *>* *0.05; *χ*^2^ test for trend) (Supplementary Fig. S3D).

### Mapping segmental aneuploidy by copy number analysis and genotyping

**Figure 3.**
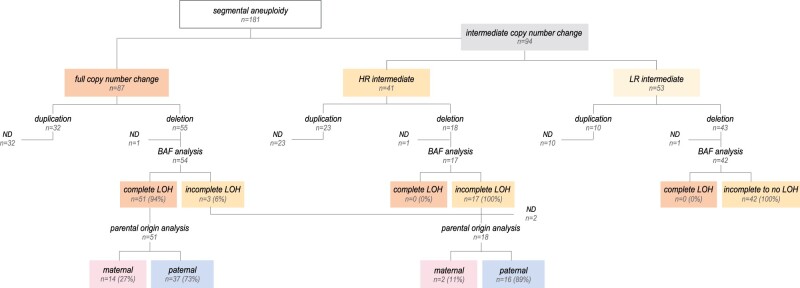
**Characterization of segmental aneuploidy according to copy number change and genotyping analysis.** Distribution of segmental aneuploidy per copy number change. Further classification of deletions by degree according to BAF and by parental origin (for copy number changes >50%). Deletions excluded from further analysis are marked as ND. BAF, b-allele frequencies; ND, not determined; LOH, loss of heterozygosity.

In total, 181 segmental aberrations were detected across 139 embryos of the cohort. Deletions (n = 116/181; 64%) were more prevalent than duplications (n = 65/181; 36%) and the size of the segmental errors varied from 10 to 197.5 Mb, with an average of 56 Mb. Not all segmental aneuploidies seemed to be present throughout the entire biopsy. Half (n = 87/181; 48%) were characterized by full copy number changes, while the other half (n = 94/181; 52%) displayed intermediate copy number changes, with a fairly even distribution between the HR (23%) and LR (29%) groups (Fig. 3). To delve deeper into these predictions, we conducted BAF analysis on the deletions (Fig. 3). Among the full copy number results, 54 out of 55 deletions retrieved a SNP diagnosis (excluding one poor quality result), most of which (94%) corresponded with complete LOH in the biopsy for the loci investigated. For HR intermediate copy number results, all 17 genotyped deletions showed incomplete LOH, suggestive of a mosaic presence in the biopsy. Consistently, none of the analysed deletions with LR intermediate copy number results demonstrated complete LOH. Two cases with intermediate copy number changes were excluded from genotyping analysis due to partial maternal contamination.

We further investigated the parental origin of deletions with copy number changes >50% (Fig. 3), and found that 73% (n = 37/51) of the deletions with complete LOH were of paternal origin. Similarly, among the analysed deletions with incomplete LOH (n = 18/20; excluding single-parent analysis (n = 1) or inconclusive results (n = 1)), 89% (n = 16/18) were of paternal origin. Thus, segmental losses occurred mainly on the paternal allele (*P *<* *0.0001, binomial test) (n = 53/69; 77%). In alignment, maternal age did not affect segmental aneuploidy rate (*P > *0.05; *χ*^2^ test for trend). Paternal age was not collected for this study and could therefore not be investigated.

### Copy number and genotyping data at the embryo level

**Figure 4.**
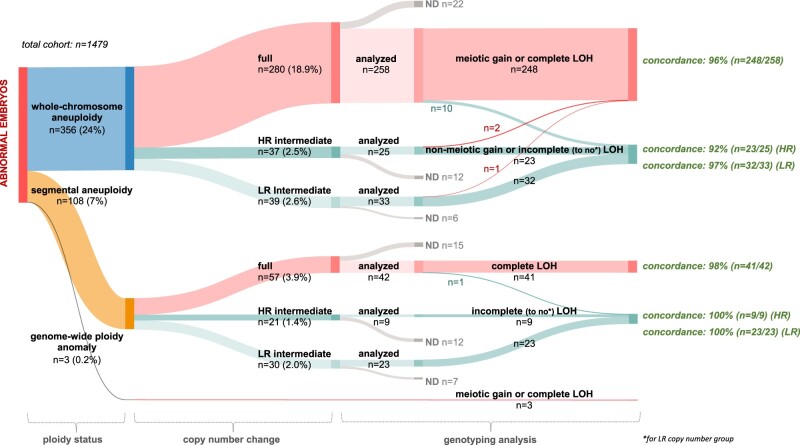
**Comprehensive assessment for embryo classification.** Sankey diagram depicting concordance between copy number change reported (second node) and genotyping analysis (third and fourth node) at the embryo level for each ploidy status (first node). In the presence of multiple chromosomal aberrations, embryos were scored according to the anomaly with the most unfavourable prognosis. Absolute numbers (n) of embryos are displayed within each node, and the width of each flow is proportional to embryo count. The percentage relative to the total embryo cohort (n = 1479) is shown in parentheses for the first two nodes (ploidy status, copy number change). Concordance (%) between copy number and genotyping analysis is depicted at the end of each respective flow. Embryos not investigated by genotyping are categorized as ND. Embryos deemed euploid are not graphed. HR, high range; LR, low range; LOH, loss of heterozygosity; ND, not determined.

Distributions of copy number changes and genotyping outcomes were charted at the embryo level (Fig. 4, Supplementary Fig. S6). In the presence of more than one whole-chromosome aneuploidy or additional segmental aneuploidy, the embryo was scored based on the whole-chromosome aneuploidy with the worst prognosis according to copy number change (full > HR intermediate > LR intermediate) and genotyping diagnosis (meiotic gain or complete LOH in biopsy > non-meiotic gain or incomplete (to no) LOH in biopsy). For embryos lacking genotype data, the rationale is summarized in Supplementary Table S1.

At the embryo level, copy number changes generally aligned well with the genotype signatures, although some embryos may require further consideration before (de)selection for transfer.

Among all embryos (n = 1479), 280 embryos (19%) harbored at least one whole-chromosome aneuploidy with full copy number changes. Of these, 258 embryos were further subjected to genotyping. For most embryos (n = 248/258; 96%), SNP signatures indeed indicated the presence of at least one meiotic trisomy and/or uniform monosomy in the biopsy, supporting deselection for clinical use. However, for a subset of embryos (n = 10/258; 4%), genotyping failed to detect any evidence of a uniform aneuploidy present. Instead, signatures of non-meiotic (or presumed mitotic) trisomy or putative mosaic monosomy in the biopsy were uncovered. From a clinical point of view, these findings may be particularly relevant for the embryos (n = 7/10) where this was a single event, and no other anomalies were present (embryo 1–3; embryo 6–9 in Table 2). Others coexisted with segmental aneuploidy (n = 2/10; embryo 5, 10) or comprised multiple discordant events within the same embryo (n = 1/10; embryo 4 in Table 2).

An additional 76 embryos (n = 5%) contained only whole-chromosome aneuploidies with HR (n = 37) or LR (n = 39) intermediate copy number changes, suggesting chromosomal mosaicism. Another 2% of embryos contained both intermediate and full copy number changes. Among embryos with HR intermediate copy number changes that were further genotyped (n = 25/37), absence of uniform aneuploidy was confirmed for most (n = 23/25; 92%). Remarkably, however, in 2 out of 25 embryos (8%) diagnosed as putative mosaic by intermediate copy number status alone, SNP signatures indicated the presence of one meiotic/uniform aneuploidy in the biopsy (embryo 12, 13 in Table 2). Surprisingly, even among one of 33 genotyped embryos diagnosed as LR mosaic by copy number, complete LOH was discovered for one of two anomalies identified (embryo 14 in Table 2).

A segmental aneuploidy with a full copy number change was identified in 57 embryos (3.9%), with only one of the analysed embryos (n = 1/42; 2%) yielding a contradictory putative mosaic diagnosis through genotyping. Interestingly, for this embryo (46, XY, del(14)(q22.3q32.33)), all other chromosomes were unaffected. Meanwhile, 3.4% of the embryos (n = 51) displayed exclusively segmental intermediate copy number changes, with absence of uniform segmental aneuploidy confirmed in those further genotyped (HR: n = 9/9; LR: n = 23/23). Notably, the rate of putative segmental mosaicism increased to 4.3% upon inclusion of embryos with additional segmental uniform copy number changes, and further to 6% when considering additional whole-chromosomal aneuploidy.

Finally, for the embryos (n = 3) diagnosed with a genome-wide anomaly, all evidence pointed to the anomaly being present in all biopsied cells or even all embryonic cells. We identified one diandric triploid embryo of MI origin, one digynic triploid embryo of MII origin, and one gynogenetic haploid embryo with complete LOH genome wide.

## Discussion

Here, we offer an extensive overview of (putative mosaic) (sub)chromosomal abnormalities observed in 1479 PGT-M embryos at the blastocyst stage. Unlike other studies focusing solely on copy number analysis, we integrated genotype information, providing enhanced insights on the status and origin of aneuploidy when examining single TE biopsies.

In our study, roughly a quarter of the blastocysts (24%) presented with whole-chromosome aneuploidy, with 19% showing a full copy number change. This aligns with aneuploidy rates from 22.9% up to 33.6% reported in PGT-M blastocysts by other studies (Li *et al.*, 2018; Hou *et al.*, 2019; Kubicek *et al.*, 2019; Tšuiko *et al.*, 2021). The higher prevalence rate (of up to 50%) reported in PGT-A blastocysts most likely reflects the influence of maternal age or underlying fertility issues in these patient groups (Franasiak *et al.*, 2014; Gruhn *et al.*, 2019).

Typically, intermediate copy number changes of a single TE biopsy are considered evidence of chromosomal mosaicism. In our study, we observed intermediate copy number changes in 7% of the embryos, most of which did not include additional full copy number results (5%). This prevalence is consistent with other observations, although huge variations, ranging from 2 to 25%, have been reported in human blastocysts (Popovic *et al.*, 2020, 2024). The substantial discrepancies in mosaicism prevalence rates may be attributed to technical variability (Capalbo *et al.*, 2017; Popovic *et al.*, 2020, 2024; Campos, 2023). For example, the diagnostic thresholds chosen for degree classification impact prevalence rates. According to Girardi *et al.* (2023), the use of wide mosaicism thresholds (i.e. 20–80%) reduces the overall PGT-A diagnostic accuracy by increasing the risk of false-positive mosaic classification and false-negative aneuploid classification compared to more stringent thresholds (30–70%). Therefore, we adopted the thresholds of 30–70% in our study. Still, even when adhering to these thresholds, variations in analytical platforms generate further variability due to differences in sensitivity and specificity (Marin *et al.*, 2021). Other factors may include TE sample size and *in vitro* culture generated variability (Popovic *et al.*, 2020). Meanwhile, concerns arise from technical artifacts that can mimic intermediate copy number changes. Such artifacts may be induced by WGA, sample contamination, incomplete cell lysis prior to amplification, or the biopsy procedure itself due to excessive laser damage, although the latter remains to our knowledge unproven (Capalbo *et al.*, 2017; Popovic *et al.*, 2020; Treff and Marin, 2021). Sifting genuine copy number changes from technical noise remains a challenge, especially when lacking the means to discern between the two. Finally, diagnosing mosaicism based on intermediate copy number changes of a single TE biopsy alone remains challenging due to sample representation. While a blastocyst-stage embryo has been estimated to have on average 200 cells (Viotti *et al.*, 2021a), embryo classification is based on a single TE biopsy of only 5–10 cells. Mathematically speaking, the complex interplay of drawing 5–10 elements from a pool of 200, without elimination, culminates in a binomial distribution. As such, a single TE biopsy may not be indicative of the rate of mosaicism of the ICM or whole blastocyst (Gleicher *et al.*, 2017), as has been demonstrated by concordance studies (Popovic *et al.*, 2018; Marin *et al.*, 2021; De Rycke *et al.*, 2022). For embryos with TE mosaicism, detection will vary by biopsy location according to the tissue distribution of euploid and aneuploid cells (Vera-Rodriguez and Rubio, 2017). Thus, even a 100% aneuploid TE biopsy cannot prove that the remaining embryonic cells are all aneuploid (Barad *et al.*, 2022). This challenge is particularly pronounced in cases of ICM/TE mosaicism, where the ICM is euploid and the TE is aneuploid. Hence, because of technical and biological variability, the degree of copy number change reported may not necessarily correlate with clinical outcome (Chuang *et al.*, 2018; Kushnir *et al.*, 2018; Victor *et al.*, 2019), although this remains a topic of debate (Spinella *et al.*, 2018; Munné *et al.*, 2020).

To enhance the confidence of copy number calls, concurrent SNP genotyping analysis has been recommended (Campos, 2023; Rana *et al.*, 2023; Janssen *et al.*, 2024). In our study, for most embryos analysed (96%), trisomies with full copy number changes were confirmed to be of meiotic origin, and monosomies with full copy number changes were accompanied by complete LOH in the TE biopsy. This validation, particularly the diagnoses of meiotic aberrations, provides reassurance that the embryo is genuinely uniformly aneuploid (Rana *et al.*, 2023), enhancing confidence for embryo deselection as they have little to no reproductive potential (Tiegs *et al.*, 2021; Capalbo *et al.*, 2022). Others reported a similar true-positive rate (97.7%) for uniform aneuploidy (classified by the same copy number thresholds) through embryo dissection (Girardi *et al.*, 2023). Nevertheless, for a notable proportion (4%) of analysed aneuploidies (and embryos), we uncovered a non-meiotic (presumed mitotic) origin for full copy number gains, and incomplete LOH in the biopsy for supposed full copy number losses, suggestive of a mosaic aneuploidy in the embryo. An even higher prevalence (16%) of mitotic aneuploidies with copy number ≥70% was reported by Handyside *et al.* (2021). The potential false-positive diagnosis of uniform aneuploidy by copy number analysis may stem from technical variability. Another explanation for full copy number gains of non-meiotic origin could be sampling bias, where the biopsy is randomly taken from a small clonal island of aneuploid cells within a mosaic embryo. The risk of inadvertently classifying embryos as uniformly aneuploid may lead to the deselection of potentially clinically viable embryos. Indeed, while mitotic errors during the first zygotic division could affect all cells (e.g. reciprocal aneuploidy due to mitotic non-disjunction) (i.e. aneuploid mosaicism), errors caused at later post-zygotic divisions (or first division by anaphase lag) induce a mixture of euploid and aneuploid cells, which may result in healthy offspring (Greco *et al.*, 2015; Lee *et al.*, 2020; Munné *et al.*, 2020; Capalbo *et al.*, 2021; Viotti *et al.*, 2021b). In fact, after more than 1000 mosaic embryo transfers, only a few cases of persistent mosaicism in pregnancies and newborns have been reported (Kahraman *et al.*, 2020; Schlade-Bartusiak *et al.*, 2022; Greco *et al.*, 2023). These successful outcomes are believed to stem from self-correction mechanisms (Bolton *et al.*, 2016; Shahbazi *et al.*, 2020; Fernandez Gallardo *et al.*, 2023), although others have suggested technical overestimation of mosaicism as a potential explanation (Campos *et al.*, 2023). Still, if transfer of putative mosaic embryos is considered, guidelines have been put in place to ensure the best clinical practices (Grati *et al.*, 2018; De Rycke *et al.*, 2022; Leigh *et al.*, 2022).

Intermediate copy number calls above 50% were mainly (92%) associated with a non-meiotic origin for trisomy or incomplete LOH for monosomy in the biopsy, thus both suggesting the absence of uniform aneuploidy in the embryo. Still, in the remaining 8% of analysed whole-chromosome aneuploidies and embryos, HR intermediate copy number deviations were contradicted by a meiotic origin or complete LOH in the biopsy, suggestive of a uniform aneuploidy in the embryo. Notably, Handyside *et al.* (2021) reported 5 out of 11 embryos diagnosed as whole-chromosome mosaic by NGS-based PGT-A to comprise maternal meiotic trisomies or monosomies upon SNP genotyping and karyomapping. Another systematic review (Marin *et al.*, 2021) stated that 28% of embryos deemed mosaic by a single biopsy were found to be uniformly aneuploid upon embryo dissection. Such discrepancies indicate a false-positive call of mosaicism by copy number emanating from technical variability or noise. This may be the particular case for the discrepancy found for chromosome 19 in our study, which is known for its high GC count posing sequencing and mapping challenges. In rare cases, intermediate copy number changes of meiotic origin or with complete LOH can be a true-positive mosaic call due to postzygotic trisomy or monosomy rescue. Although, such correction often co-exists with uniparental disomy (UPD), and evidence of such mechanisms remains scarce (Gueye *et al.*, 2014). In our cohort, the combination of LR monosomy and complete LOH may be indicative of monosomy rescue in a fraction of cells. Furthermore, our genotyping data unveiled partial maternal contamination in four aneuploidies with intermediate copy number within two embryos. Contamination can skew copy number changes from full to intermediate, potentially leading to misdiagnosis. Overall, if these embryos are in fact uniformly aneuploid, transferring them under the premise of mosaicism could cause patients harm, given the close-to-zero reproductive potential of uniform aneuploid embryos (Tiegs *et al.*, 2021; Capalbo *et al.*, 2022).

Genotyping analysis adds a second dimension to the one-dimensional Log_2_ copy number ratios. Still, our results should be interpreted with caution. Here, PGT-A results are suggestive of mosaicism when observing intermediate (or even full) copy number changes without evidence of a meiotic origin or complete LOH in the biopsy. Based on this reasoning, the prevalence of putative chromosomal mosaicism stands at 5.7% in our study, which is fairly similar to the mitotic aneuploidy rate (2%) observed by Rana *et al.* (2023). Nonetheless, we refrain from interpreting the absence of meiotic origin as conclusive evidence of a mitotic error. Our analysis is based on single TE biopsies, lacking embryo disaggregation to provide concrete evidence of a mitotic error, nor did we process heterozygous BAF values to determine exact mosaicism levels in the biopsy (Zamani Esteki *et al.*, 2015; Verdyck *et al.*, 2022). Thus, the prevalence of putative mosaicism might still be overestimated, and a considerable portion may actually be euploid, as seen by re-biopsy studies (Marin *et al.*, 2021). This concern may be particularly relevant for copy number changes below 50%. On the other hand, we also cannot definitively rule out the presence of mosaicism beyond a single euploid TE biopsy without complete embryo dissection. While not researched here, others have reported false-negative rates below 4–8% (Popovic *et al.*, 2020; Marin *et al.*, 2021). Finally, relying on a single TE biopsy restricts the analysis of the cell-division origin of monosomies. While LOH in a single biopsy might indicate a meiotic origin, a mitotic error cannot be entirely ruled out due to sampling bias. Instead, genotype data of monosomies is relevant only to the biopsy itself. Hence, to evaluate the true-positive and false-positive rates of mosaicism detection, embryo dissection and ideally single-cell analysis is warranted. Therefore, in our context, rather than using genotype analysis as a selection tool for putative mosaic embryos, it should serve as a deselection tool by identifying meiotic aberrations, aiming to minimize adverse clinical outcomes (Janssen *et al.*, 2024; Popovic *et al.*, 2024). In fact, some experts even argue that mosaicism should not be reported as standard but should be considered a finding of unknown significance, given that mosaicism cannot always be accurately assessed in TE biopsies (especially with copy number analysis alone), and its clinical relevance remains unclear (Treff and Marin, 2021; Campos, 2023).

Our parent-of-origin study confirmed that whole-chromosome aneuploidy mainly arises during female meiosis, with its incidence rising with maternal age. The established error-prone nature of female meiosis is driven by multiple factors, including the prolonged resting phase in prophase I, as indicated by the predominance of maternal MI errors in our study and others (Kubicek *et al.*, 2019; Konstantinidis *et al.*, 2020; Tšuiko *et al.*, 2020). Although not studied here, premature separation of sister chromatids due to age-related loss of cohesin is hypothesized as the primary cause for maternal age-related MI errors (Verdyck *et al.*, 2023). We further confirmed the predisposition of meiotic errors in acrocentric and short chromosomes, predominantly in chromosome 16 and 22 (Tšuiko *et al.*, 2021). In contrast, for intermediate copy number changes (of non-meiotic origin) suggesting mitotic aneuploidy, no significant parental preference was observed, consistent with other research (McCoy *et al.*, 2015; Tšuiko *et al.*, 2021). We further verified that (presumed) mitotic aneuploidy is not influenced by maternal age (McCoy *et al.*, 2015; Munné and Wells, 2017), nor by chromosome size.

Segmental aneuploidy too occurs at a considerable frequency in preimplantation embryos. Incidences of 5.6–15.6% have been reported at the blastocyst stage (Babariya *et al.*, 2017; Escribà *et al.*, 2019; Girardi *et al.*, 2020), either exclusively or in conjunction with whole-chromosome aneuploidy, which is consistent with our data (exclusive: 7%, total: 9%). Unlike whole-chromosomal aneuploidy, segmental aneuploidy has been described to arise mainly post-zygotically (Babariya *et al.*, 2017; Girardi *et al.*, 2020) and on paternal chromosomes, implicating sperm DNA damage in its formation (Kubicek *et al.*, 2019; Tšuiko *et al.*, 2021). We observed a similar subclassification, identifying a predominance on the paternal allele (77%) and a high, albeit not statistically significant, percentage of putative mosaic segmental aneuploidies (52%) according to copy number changes, closely matching genotyping data (94–100%). While valuable, we note that duplications could not be evaluated for genotyping analysis. Additionally, mosaicism rates were possibly underestimated due to the larger diagnostic reporting size (>20 Mb) set for copy number changes under 50%.

Finally, through genotyping analysis, we identified gynogenetic haploidy at the blastocyst stage at a low frequency (0.1%). UPD was absent in our cohort. This was not entirely unexpected, given its exceedingly rare prevalence (0.06%) reported by others (Gueye *et al.*, 2014). Triploidy has also been documented at a very low frequency in blastocysts (<1–1.7%) (McCoy *et al.*, 2015; Tšuiko *et al.*, 2021). Here, the prevalence amounted to 0.1%, from identifying one diandric triploid blastocyst of MI origin and one digynic triploid blastocyst of MII origin. While valuable, the reported haploidy/triploidy rates may be an underestimation for the general population as time-lapse imaging with pronuclei (PN) screening is performed in our centre, indicating that the observed ploidy anomalies were most likely not picked up during PN checks.

In conclusion, integrating genotyping analysis alongside copy number analysis could provide a more comprehensive assessment of the embryo’s genetic makeup, within and beyond the single TE biopsy. By identifying meiotic aberrations, we underscore the potential value of genotyping analysis as a deselection tool, ultimately striving to reduce adverse clinical outcomes. In the future, reliable identification of mitotic aneuploidy may hold promise for enhancing the diagnostic accuracy of chromosomal mosaicism. Further large-scale validation studies for genotype-based mosaicism detection are warranted, along with diligent follow-up of pregnancies following mosaic embryo transfer.

## Supplementary Material

hoae056_Supplementary_Data

## Data Availability

Summary data underlying this article are available within the article and its supplementary information files. Specific datasets or genetic profiles are not publicly available to protect individual patient confidentiality but are available from the corresponding author on reasonable request. Hopla (v1.0.6) is freely available at GitHub (https://github.com/CenterForMedicalGeneticsGhent/Hopla) and ViVar is accessible at https://vivar.cmgg.be.
