# Supplementary material for: Aligning genotyping and copy number data in single trophectoderm biopsies for aneuploidy prediction: uncovering incomplete concordance
Source: Hum Reprod Open. 2024 Sep 18;2024(4):hoae056. doi: 10.1093/hropen/hoae056 (PMC11461285; doi:10.1093/hropen/hoae056)
Supplement: hoae056_Supplementary_Data [file hoae056_supplementary_data.zip › Supplementary Table S1.pdf]

Supplementary Table S1: Rationale for undetermined genotyping diagnosis at the embryo level

undetermined SNP genotyping diagnosis	whole-chromosome aneuploidy			segmental aneuploidy		
	full CN change (n)	HR intermediate CN change (n)	LR intermediate CN change (n)	full CN change (n)	HR intermediate CN change (n)	LR intermediate CN change (n)
**total**	**22**	**12**	**6**	**15**	**12**	**7**
single-parent processing[a]	5	1	-	-	-	-
inconclusive result	1	-	1	-	-	-
absence crossover in trisomic chromosome[b]	5	3	3	-	-	-
partial maternal contamination	-	1	1	-	-	-
complex aneuploidy[c]	11	7	1	-	-	-
duplication[d]	-	-	-	15	12	7

Data represent the total number of embryos in each group.
[a]Genotyping is contingent on the availability of DNA of both parents.
[b]Distinction between meiotic II and non-meiotic errors is only feasible when a crossover event has occurred in the trisomic chromosome.
[c]Complex abnormal embryos affected by five or more aberrations are not subjected to genotyping analysis.
[d] Duplications are not investigated due to technological limitations.
CN: copy number; HR: high-range; LR: low-range.
