# Supplementary material for: Aligning genotyping and copy number data in single trophectoderm biopsies for aneuploidy prediction: uncovering incomplete concordance
Source: Hum Reprod Open. 2024 Sep 18;2024(4):hoae056. doi: 10.1093/hropen/hoae056 (PMC11461285; doi:10.1093/hropen/hoae056)
Supplement: hoae056_Supplementary_Data [file hoae056_supplementary_data.zip › Supplementary Figures.pdf]

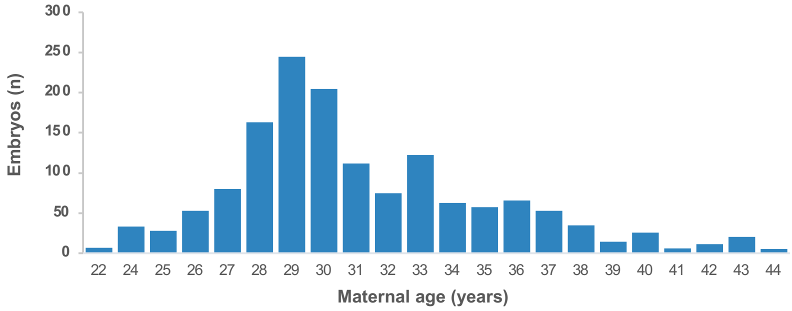

Supplementary Figure S1: Distribution of examined embryos by maternal age. The absolute number of embryos is plotted on the y-axis and the maternal age (expressed in years) is plotted on the x-axis.

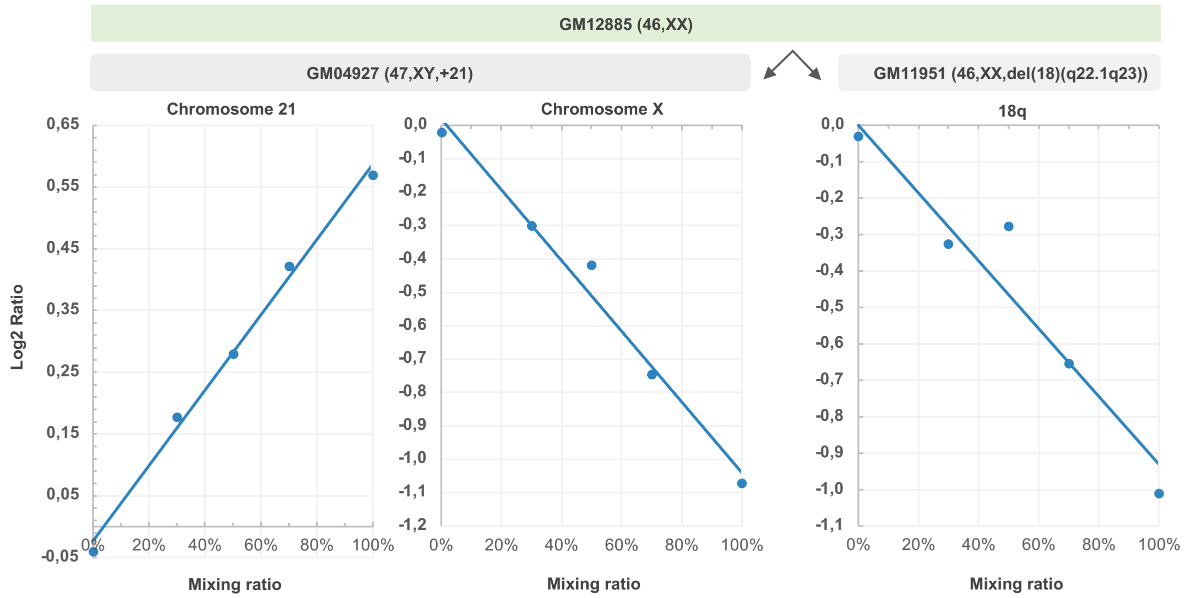

Supplementary Figure S2. Validation GENType for intermediate copy number detection through cell mixing experiments. Mosaicism was simulated by combining aneuploid cells with euploid cells in different ratios: 0:10 (0%), 3:7 (30%), 5:5 (50%), 7:3 (70%), 10:0 (100%). Per mixing ratio, the average of the collected Log_2 values is presented. On the left, results are shown for the cell mixing experiments of aneuploid cell line GM04927 (47,XY,+21) with euploid cell line GM12885 (46,XX). Both chromosome 21 (left) and chromosome X (i.e., XX/XY ratio in the middle) were investigated. On the right, the results are shown for cell line GM11951 (46,XX,del(18)(q22.1q23)), carrying a 15Mb terminal deletion. Overall, a linear correlation was observed between the LogR value (y-axis) and the number of aneuploid cells within the biopsy (x-axis). Intermediate copy number changes can be deducted for the corresponding ratios.

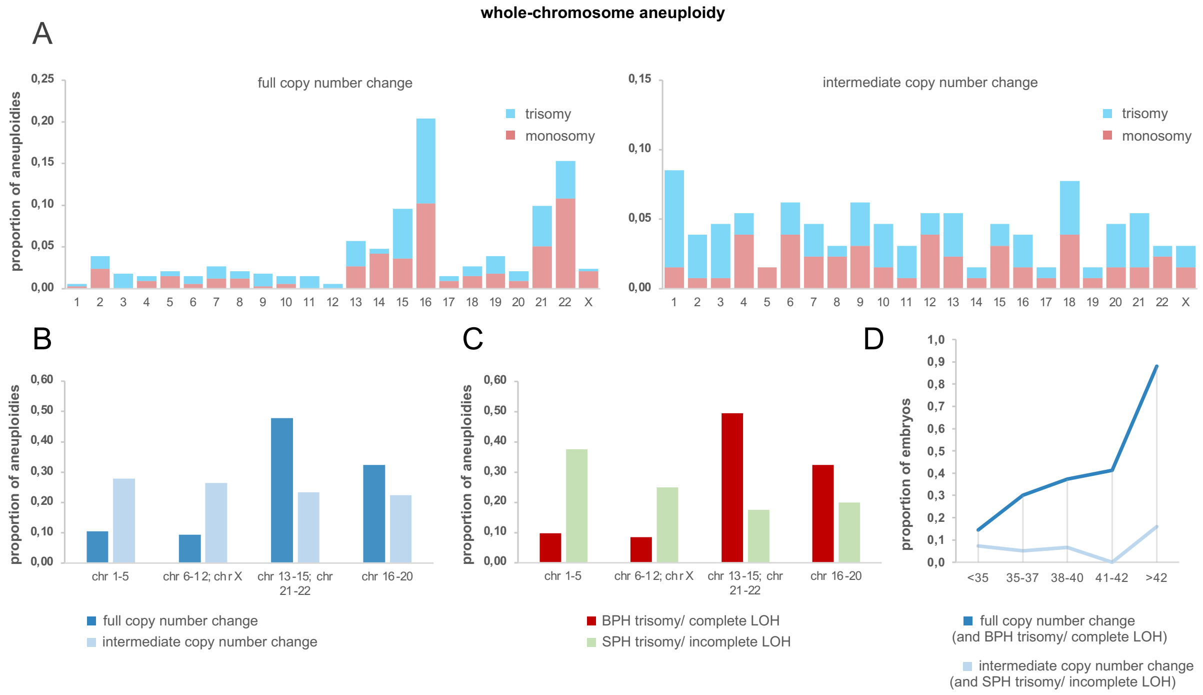

Supplementary Figure S3. Distribution of whole-chromosome aneuploidy by chromosome type and maternal age. A) Proportion of whole-chromosome aneuploidy (y-axis) observed per chromosome (x-axis) for full copy number changes (on the left) and HR or LR intermediate copy number changes (on the right). Corresponding trisomy rate and monosomy rate per chromosome are shown in blue and red, respectively. **B)** Correlation between aneuploidy rate (y-axis) and chromosome classification by length (x-axis) (large (chr 1-5), medium-sized (chr 6-12 and chrX), acrocentric (chr 13-15 and chr 21-22) and short (chr 16-20) chromosomes). Distribution is shown for full copy number changes (dark blue, $P < 0{,}0001$; $\chi 2$ test) and intermediate copy number changes (light blue, $P > 0{,}05$; $\chi 2$ test). **C)** Genotype signatures (observed for copy number values >50%) in relation to chromosome length. BPH trisomy along with monosomy characterized by complete LOH are shown in red ($P < 0{,}0001$; $\chi 2$ test), while SPH trisomy and monosomy with incomplete LOH are represented in green ($P > 0{,}05$; $\chi 2$ test). **D)** Influence of maternal age (x-axis) on the prevalence of embryos (y-axis) carrying whole-chromosome aneuploidy. Distribution is depicted for full copy number changes (with corresponding genotype signatures, if available) (dark blue, $P < 0{,}0001$; $\chi 2$ test for trend) and for intermediate copy number changes (with corresponding genotype signatures, if available) (light blue, $P > 0{,}05$; $\chi 2$ test for trend). The latter include all embryos with intermediate copy number changes, regardless of whether additional full copy number changes are present. HR: high-range; LR: low-range; BPH: both parental homologs; LOH: loss of heterozygosity; SPH: single parental homolog.

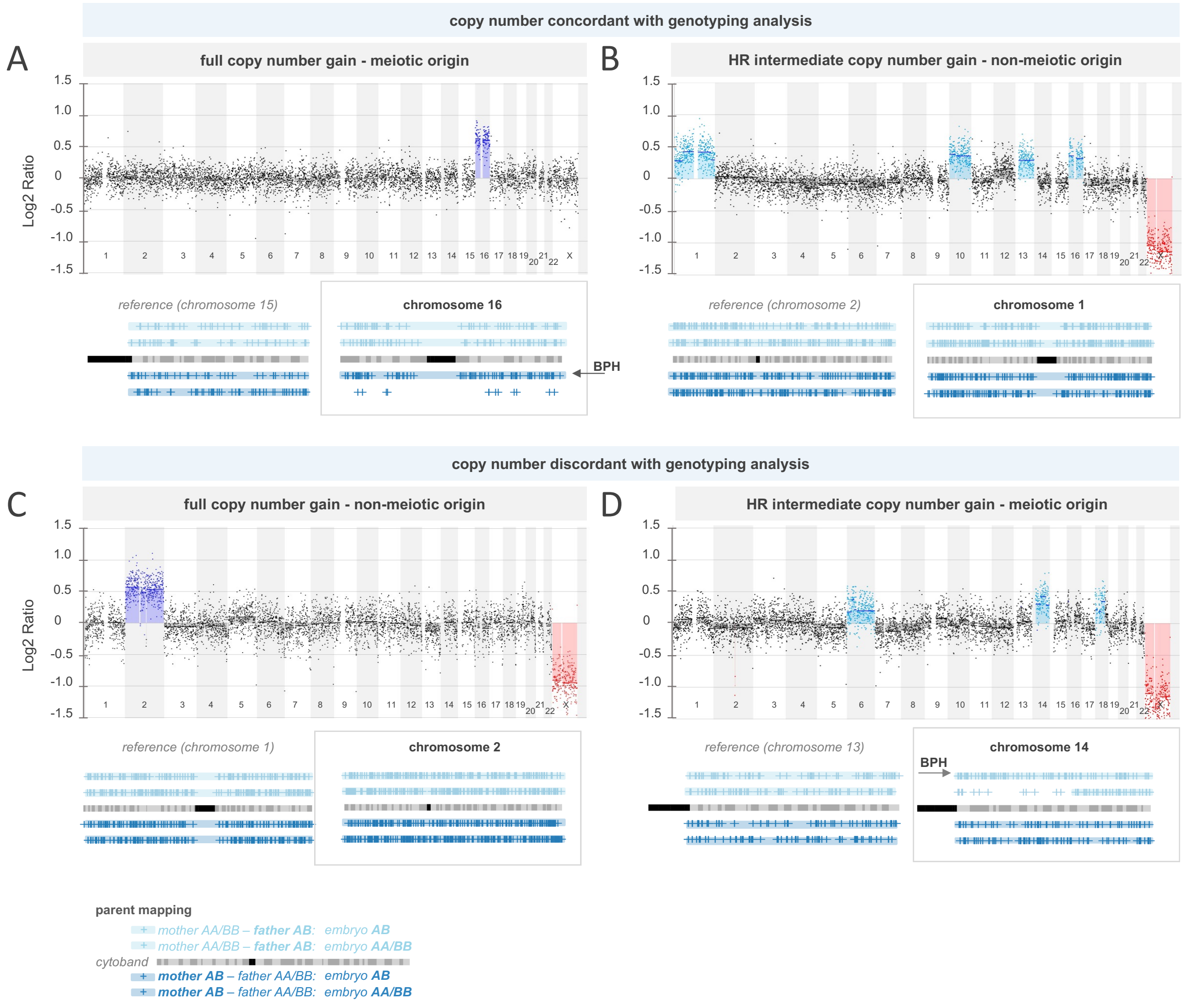

Supplementary Figure S4. Comparison of copy number change with meiotic division of origin for trisomy. On top, examples are given of trisomies characterized by copy number changes consistent with their genotyping diagnosis (i.e., full copy number = meiotic origin; intermediate copy number = non-meiotic or presumed mitotic origin). Conflicting results are shown below. For each example, we show the genome-wide copy number profile at the top. Full copy number changes (i.e., >70%) are depicted in dark blue, copy number changes in the intermediate range (i.e., 30%-70%) are depicted in light blue. Below the copy number profile, the meiotic division of origin of the investigated trisomy is illustrated through parent mapping profiling (framed, right side). A reference parent mapping profile of an adjacent chromosome deemed euploid from the same embryo is provided for comparison (left side). Parent mapping profiling was previously explained in detail by De Witte *et al.* (2022). Briefly, SNP genotypes (illustrated as crosses) of the embryo are distributed over 4 tracks (legend lower left). Crosses above the cytoband represent informative SNPs of the father (light blue), while crosses below the cytoband represent informative SNPs of the mother (dark blue). For each category, the top and bottom tracks represent SNPs for which the embryo is heterozygous and homozygous, respectively. By studying the distribution of the SNPs, the meiotic division of origin can be identified. Meiotic trisomies are identified by the presence of BPH of a single parent across the centromere (meiotic I error) or other regions of the chromosome (meiotic II error), showing predominantly heterozygous SNPs (i.e., 3 tracks instead of 4) for the respective parent at the corresponding sites. Non-meiotic errors possess an extra copy of SPH from the respective parent with no change in the distribution of

the SNPs (i.e., standard 4-track). For visualization purposes, the observed tracks were highlighted. **A)** The full copy number changes of trisomy 16 were confirmed by a maternal meiotic I origin. **B)** The HR intermediate copy numbers of trisomy 1, 10, 13, 16 were consistent with a non-meiotic (or presumed mitotic) origin (here only displayed for trisomy 1). **C)** The full copy number observed for trisomy 2 was of non-meiotic (or presumed mitotic) origin. **D)** Trisomy 14, diagnosed with intermediate copy number changes, was found to be a meiotic I error of paternal origin. BPH: both parental homologs; SPH: single parental homolog; HR: high-range.

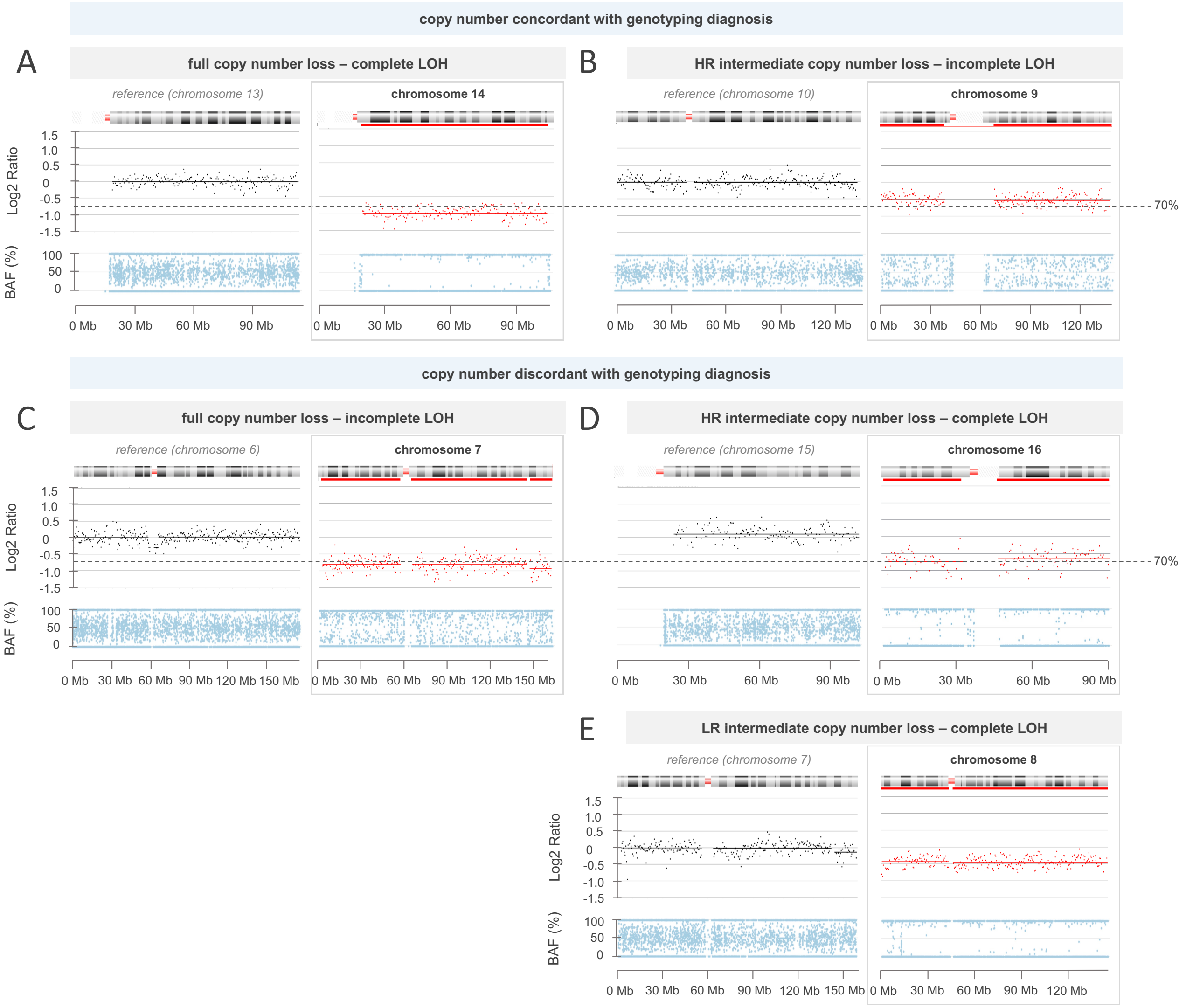

Supplementary Figure S5. Comparison of copy number change with BAF values for monosomy. For each example, we show the copy number profile (Log$_2$ ratio) (top) and the BAF profiles of the chromosome (bottom). Monosomies with copy number changes exceeding 30% are depicted in red. A dashed line is included to discern between full (>70%) and HR intermediate copy number changes (50%-70%). BAF profiles were examined for complete LOH (i.e., solely BAF values of 0 and 100%) or incomplete LOH (i.e., BAF values between 0-50% and 50-100%). Profiles of the investigated monosomies are framed and displayed on the right, while reference profiles (of an adjacent euploid chromosome) from the same embryo are provided on the left. Concordant results between copy number analysis and BAF analysis are shown on top. Monosomy 14 is identified as uniformly aneuploid by both datasets (i.e., full copy number changes with complete LOH) (A), while monosomy 9 is consistently reported as putative mosaic (i.e., intermediate copy number changes with incomplete LOH) (B). In the lower section, inconsistent results are shown. Monosomy 7 with a copy number just within the full range displays BAF values between 0-50% and 50-100% (i.e., incomplete LOH) (C). Conversely, complete LOH is observed for monosomy 16 (D) and monosomy 8 (E), despite having copy number changes in the HR intermediate (D) and LR intermediate (E) categories, respectively. BAF: b-allele frequencies; HR: high-range; LR: low-range; LOH: loss of heterozygosity.

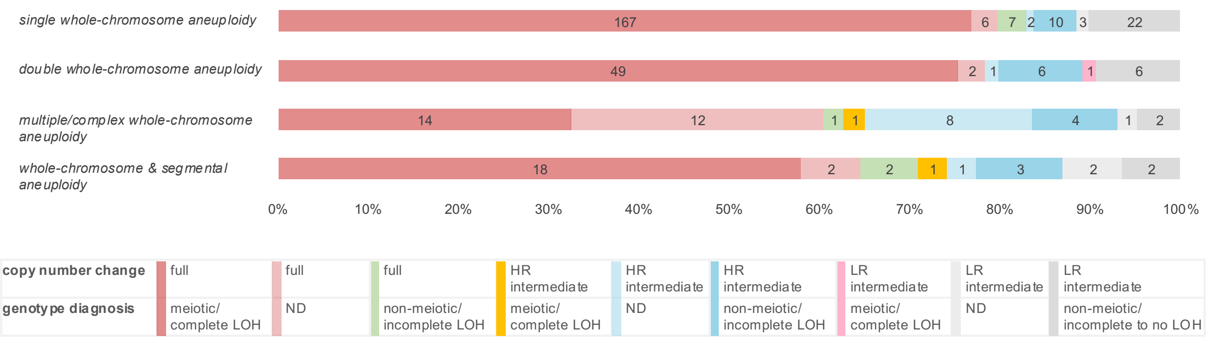

Supplementary Figure S6. Comprehensive classification of embryos with various counts of whole-chromosome aneuploidy. Classification of embryos with single, double, and multiple or complex whole-chromosome aneuploidy or those combined with segmental aneuploidy (left y-axis), based on copy number reported and genotyping diagnosis. Absolute numbers (n) are illustrated in the middle of each bar. The length of the bar represents the percentage of the embryos within each category. Embryos displaying solely segmental aneuploidy are not graphed. LOH: loss of heterozygosity; HR: high-range; LR: low-range.
